# Prevalence of pathogenic germline variants detected by multigene sequencing in unselected Japanese patients with ovarian cancer

**DOI:** 10.18632/oncotarget.22733

**Published:** 2017-11-28

**Authors:** Akira Hirasawa, Issei Imoto, Takuya Naruto, Tomoko Akahane, Wataru Yamagami, Hiroyuki Nomura, Kiyoshi Masuda, Nobuyuki Susumu, Hitoshi Tsuda, Daisuke Aoki

**Affiliations:** ^1^ Department of Obstetrics and Gynecology, Keio University School of Medicine, Tokyo, Japan; ^2^ Department of Human Genetics, Graduate School of Biomedical Sciences, Tokushima University, Tokushima, Japan; ^3^ Department of Obstetrics and Gynecology, International University of Health and Welfare, Chiba, Japan; ^4^ Department of Basic Pathology, National Defense Medical College, Saitama, Japan

**Keywords:** ovarian cancer, germline, pathogenic variant, multigene panel, Japanese women

## Abstract

Pathogenic germline *BRCA1*, *BRCA2* (*BRCA1/2*), and several other gene variants predispose women to primary ovarian, fallopian tube, and peritoneal carcinoma (OC), although variant frequency and relevance information is scarce in Japanese women with OC. Using targeted panel sequencing, we screened 230 unselected Japanese women with OC from our hospital-based cohort for pathogenic germline variants in 75 or 79 OC-associated genes. Pathogenic variants of 11 genes were identified in 41 (17.8%) women: 19 (8.3%; *BRCA1*), 8 (3.5%; *BRCA2*), 6 (2.6%; mismatch repair genes), 3 (1.3%; *RAD51D*), 2 (0.9%; *ATM*), 1 (0.4%; *MRE11A*), 1 (*FANCC*), and 1 (*GABRA6*). Carriers of *BRCA1/2* or any other tested gene pathogenic variants were more likely to be diagnosed younger, have first or second-degree relatives with OC, and have OC classified as high-grade serous carcinoma (HGSC). After adjustment for these variables, all 3 features were independent predictive factors for pathogenic variants in any tested genes whereas only the latter two remained for variants in *BRCA1/2*. Our data indicate similar variant prevalence in Japanese patients with OC and other ethnic groups and suggest that HGSC and OC family history may facilitate genetic predisposition prediction in Japanese patients with OC and referring high-risk patients for genetic counseling and testing.

## INTRODUCTION

Primary ovarian, fallopian tube, and peritoneal carcinoma (OC) remains the most lethal gynecological malignancy [1], because most women with OC are diagnosed at an advanced stage. Although most cases of OC are sporadic, at least 10% of patients with OC have a genetic predisposition [2]. Identifying individuals at risk for hereditary cancer syndrome with OC predisposition enables targeted prevention, early detection, and effective treatment of this disease.

Hereditary breast and ovarian cancer (HBOC) and Lynch syndrome comprise two common hereditary cancer syndromes associated with increased likelihood of OC; these are caused by pathogenic germline variants in *BRCA1* and *BRCA2* (*BRCA1/2*) and mismatch repair (MMR) genes including *MLH1*, *MSH2*, *MSH6*, and *PMS2*, respectively. Pathogenic germline variants in other genes (e.g., genes from the BRCA-Fanconi-anemia pathway, such as *BRIP1* and *RAD51*) have also been associated with an elevated risk of OC [3, 4]. Analysis of these genes may therefore be useful for identifying individuals with an OC predisposition for effective prevention strategies, early diagnosis, and prediction of therapeutic efficacy such as for poly(ADP-ribose) polymerase inhibitors (PARPi). Although recent findings have indicated that approximately 10–20% of OC cases are associated with pathogenic germline variants in cancer susceptibility genes [4–7], most patients in those studies were Caucasian. Conversely, the frequency of pathogenic germline variants in cancer susceptibility genes (including *BRCA1*/*2*) in unselected patients with OC is largely unknown in the Japanese population. In *BRCA1*/*2*, for example, data on the frequency of pathogenic germline variants may be important for estimating the rate of patients with OC who may benefit from treatment with a PARPi before the drug is approved in Japan. Furthermore, pathogenic germline *BRCA1*/*2* variants are associated with high-grade serous carcinoma (HGSC), which is less frequently found in East Asian populations (including Japanese) compared with Caucasian populations [8]; thus, the frequency of pathogenic germline variants in Japanese patients with OC may differ from that in Caucasians.

Accordingly, this study was conducted to determine the prevalence of pathogenic germline variants of OC-associated genes including known predisposing genes in an unselected group of 230 Japanese patients with OC, using next-generation sequencing (NGS)-based comprehensive targeted panel sequencing (TPS) for 75 or 79 candidate genes. The putative pathogenic variants were interpreted based on a database from Myriad Genetics [9] and a well-evaluated classification of variants in guidelines from the American College of Medical Genetics and Genomics (ACMG) [10].

## RESULTS

### Description of the study population

In total, 230 germline DNA samples from unselected patients with OC that had been stored in the Keio Women's Health Biobank (KWB) in Keio University School of Medicine (Tokyo, Japan) were analyzed by targeted resequencing, using panels of 75 or 79 candidate OC-associated genes (Supplementary Table 1). The clinicopathological characteristics of the patients are shown in Table 1. The median (range) age at diagnosis was 54 (27–87) years. Histological subtyping revealed that 74/230 (32.2%) and 71/230 (30.9%) cases were HGSC and clear cell carcinoma, respectively.

**Table 1:** Characteristics of the patients included in this study

Characteristic	*n*	%
Total	230	
Age, years		
median (range)	54 (27–87)	
<40	18	0.4
40–49	70	30.4
50–59	64	27.8
60–69	52	22.6
70–79	22	9.6
≥80	4	1.7
Disease site		
Ovary	217	94.3
Peritoneal	5	2.2
Fallopian tube	8	3.5
Histological subtype		
High-grade serous	74	32.2
Low-grade serous	3	1.3
Endometrioid	58	25.2
Clear cell	71	30.9
Mucinous	18	7.8
Others	6	2.6
Stage		
I	107	46.5
II	25	10.9
III	80	34.8
IV	18	7.8
Personal history (*n* = 180)		
Proband had breast cancer	6	3.3
Family history (*n* = 180)		
First or second-degree relative had breast cancer	26	14.4
First or second-degree relative had ovarian cancer	13	7.2
Pathogenic germline variant		
*BRCA1*	19	8.3
*BRCA2*	8	3.5
*MLH1*	1	0.4
*MSH2*	1	0.4
*MSH6*	2	0.9
*PMS2*	2	0.9
*RAD51D*	3	1.3
*ATM*	2	0.9
*MRE11A*	1	0.4
*FANCC*	1	0.4
*GABRA2*	1	0.4

### Pathogenic germline variants identified in the cohort of patients with OC

For all coding exons of the 75 or 79 genes selected for study, single-nucleotide variants (SNVs), short insertions/deletions (InDels), and copy-number variations (CNVs) were simultaneously detected using data from TPS. Pathogenic or likely pathogenic germline variants in the tested genes, as classified by the ACMG guidelines, are listed in Table 2 [10]. Of 230 patients, 19 (8.3%) and 8 (3.5%) cases carried germline *BRCA1* and *BRCA2* pathogenic variants, respectively, of which only 1 had a gross deletion covering more than 1 exon in *BRCA1*. No variants of uncertain significance in the *BRCA1/2* genes were detected in our analysis, based on the Myriad Genetics database [9]. In addition, 6 patients (2.6%) carried pathogenic variants of MMR genes, which may increase the risk of developing Lynch syndrome-related tumors, including 1 in *MLH1*, 1 in *MSH2*, 2 in *MSH6*, and 2 in *PMS2*. Furthermore, various pathogenic variants were found in several other genes including 3 (1.3%) in *RAD51D*, 2 (0.9%) in *ATM*, 1 (0.4%) in *MRE11A*, 1 in *FANCC*, and 1 in *GABRA6*. In total, 41/230 (17.8%) women with OC had pathogenic germline variants in 11 OC-associated genes.

**Table 2:** List of pathogenic germline variants in tested genes and clinicopathological features of patients with OC

	Patient ID	Age	Gene	Refseq ID	Nucleotide change	Amino acid change	Disease site	Other cancers^a^	Histological subtype	Stage	Personal history of breast cancer	Breast cancer patients in family^b^	Ovarian cancer patients in family^b^
1	OC102	39	*BRCA1*	NM_007294.3	c.4073_4074del	p.(Glu1358Alafs^*^9)	ovary	none	high-grade serous	III	No	0	1
2	OC088	59	*BRCA1*	NM_007294.3	c.2860_2864del	p.(Leu954Ilefs^*^15)	ovary	none	high-grade serous	III	No	0	1
3	OC386	42	*BRCA1*	NM_007294.3	c.188T>A	p.(Leu63^*^)	ovary	none	high-grade serous	III	No	0	0
4	OC315	36	*BRCA1*	NM_007294.3	c.5095C>T	p.(Arg1699Trp)	ovary	none	high-grade serous	III	No	0	0
5	OC677	49	*BRCA1*	NM_007294.3	c.4870_4871insA	p.(Gly1624Glufs^*^3)	ovary	none	high-grade serous	II	No	0	0
6	OC252	42	*BRCA1*	NM_007294.3	c.188T>A	p.(Leu63^*^)	ovary	none	clear cell	I	No	0	0
7	OC613	46	*BRCA1*	NM_007294.3	c.188T>A	p.(Leu63^*^)	ovary	none	high-grade serous	III	No	0	0
8	OC253	27	*BRCA1*	NM_007294.3	c.2188G>T	p.(Glu730^*^)	ovary	none	endometrioid	IV	No	1	0
9	OC600	50	*BRCA1*	NM_007294.3	c.456_457del	p.(Ser153Cysfs^*^5)	ovary	none	high-grade serous	II	No	2	1
10	OC672	54	*BRCA1*	NM_007294.3	c.4065_4068del	p.(Asn1355Lysfs^*^10)	ovary	none	high-grade serous	IV	No	1	0
11	OC644	44	*BRCA1*	NM_007294.3	c.2111del	p.(Asn704Metfs^*^32)	ovary	none	others	IV	No	1	0
12	OC070	49	*BRCA1*	NM_007294.3	c.3442del	p.(Glu1148Argfs^*^7)	ovary	CC	high-grade serous	III	No	0	0
13	OC268	48	*BRCA1*	NM_007294.3	c.188T>A	p.(Leu63^*^)	peritoneal	none	high-grade serous	IV	No	0	0
14	OC328	47	*BRCA1*	NM_007294.3	c.2800C>T	p.(Gln934^*^)	fallopian tube	none	high-grade serous	III	No	0	0
15	OC691	48	*BRCA 1*	NM_007294.3	BRCA1 del (ex20–23e)	gross deletion	ovary	BC	high-grade serous	II	Yes	1	2
16	OC080	40	*BRCA1*	NM_007294.3	c.3627dup	p.(Glu1210Argfs^*^9)	ovary	none	high-grade serous	IV	No	NA	NA
17	OC002	42	*BRCA1*	NM_007294.3	c.2800C>T	p.(Gln934^*^)	ovary	none	high-grade serous	III	No	NA	NA
18	OC323	51	*BRCA1*	NM_007294.3	c.2800C>T	p.(Gln934^*^)	ovary	none	high-grade serous	III	No	NA	NA
19	OC154	39	*BRCA1*	NM_007294.3	c.4487C>A	p.(Ser1496^*^)	ovary	none	high-grade serous	III	No	NA	NA
20	OC634	56	*BRCA2*	NM_000059.3	c.1813del	p.(Ile605Tyrfs^*^9)	ovary	none	high-grade serous	III	No	1	2
21	OC681	77	*BRCA2*	NM_000059.3	c.1125del	p.(Phe376Leufs^*^23)	ovary	none	high-grade serous	II	No	0	0
22	OC622	58	*BRCA2*	NM_000059.3	c.5560_5561del	p.(Val1854Phefs^*^3)	ovary	none	high-grade serous	III	No	0	0
23	OC629	50	*BRCA2*	NM_000059.3	c.3599_3600del	p.(Cys1200^*^)	ovary	none	high-grade serous	IV	No	0	0
24	OC004	61	*BRCA2*	NM_000059.3	c.6952C>T	p.(Arg2318^*^)	ovary	none	high-grade serous	IV	No	0	0
25	OC025	70	*BRCA2*	NM_000059.3	c.7482_7483insCC	p.(Ile2495Profs^*^30)	ovary	PC	endometriod	III	No	0	0
26	OC047	67	*BRCA2*	NM_000059.3	c.5576_5579del	p.(Ile1859Lysfs^*^3)	ovary	BC	clear cell	I	Yes	0	0
27	OC076	69	*BRCA2*	NM_000059.3	c.6656C>G	p.(Ser2219^*^)	ovary	none	high-grade serous	III	No	NA	NA
28	OC271	36	*MLH1*	NM_000249.3	c.2052T>G	p.(Tyr684^*^)	ovary	BC + CRC	endometrioid	II	Yes	NA	NA
29	OC490	36	*MSH2*	NM_000251.2	c.2362dup	p.(Thr788Asnfs^*^11)	ovary	CRC + PC + UC	high-grade serous	I	No	0	0
30	OC216	55	*MSH6*	NM_000179.2	c.3172+1G>T		ovary	UC	high-grade serous	I	No	NA	NA
31	OC096	44	*MSH6*	NM_000179.2	c.2150_2153del	p.(Val717Alafs^*^18)	ovary	UC + CC	endometrioid	III	No	0	1
32	OC313	61	*PMS2*	NM_000535.5	c.2276-1G>C		ovary	none	high-grade serous	III	No	NA	NA
33	OC662	69	*PMS2*	NM_000535.5	c.51_55del	p.(Ile18Serfs^*^34)	peritoneal	none	high-grade serous	III	No	0	0
34	OC174	44	*RAD51D*	NM_002878.3	c.270_271dup	p.(Lys91Ilefs^*^13)	ovary	none	clear cell	I	No	0	0
35	OC667	44	*RAD51D*	NM_002878.3	c.270_271dup	p.(Lys91Ilefs^*^13)	peritoneal	none	high-grade serous	III	No	0	0
36	OC670	64	*RAD51D*	NM_002878.3	c.270_271dup	p.(Lys91Ilefs^*^13)	fallopian tube	none	high-grade serous	III	No	1	0
37	OC678	48	*ATM*	NM_000051.3	c.7091del	p.(Ala2364Glufs^*^2)	ovary	none	high-grade serous	I	No	0	0
38	OC343	51	*ATM*	NM_000051.3	c.1258del	p.(Ile420Tyrfs^*^17)	ovary	none	mucinous	I	No	0	0
39	OC646	73	*FANCC*	NM_000136.2	c.406C>T	p.(Gln136^*^)	ovary	none	high-grade serous	IV	No	NA	NA
40	OC367	45	*GABRA6*	NM_000811	c.241del	p.(Val81Phefs^*^12)	ovary	none	others	III	No	0	0
41	OC058	63	*MRE11A*	NM_005591.3	c.659+1G>A		ovary	none	clear cell	III	No	NA	NA

^a^BC, breast cancer; CC, cervical cancer; CRC, colorectal cancer; PC, pancreatic cancer; UC, uterine cancer.

^b^Family history of cancer, including first- or second-degree relatives. NA, not available.

### Association between clinicopathological characteristics and pathogenic germline variants

Clinicopathological features for patients with pathogenic germline variants are described in Table 2 and Table 3. Patients with OC and pathogenic germline variants of *BRCA1/2* or any other tested genes were diagnosed at a younger age compared with patients lacking pathogenic variants in those genes.

**Table 3:** Correlation between patient characteristics and pathogenic germline variants in *BRCA1/2* or any tested genes among 230 patients with OC^a^

Clinicopathological features	*n*	Pathogenic *BRCA1*/*2* mutation		*P* value^b^	Pathogenic mutations in any tested gene		*P* value^b^
		Positive	Negative		Positive	Negative	
Age							
<55	117	20	97	**0.0132**	28	89	**0.0159**
≥55	113	7	106		13	100	
Histologic subtype^c^							
HGSC	74	22	52	**<0.0001**	30	44	**<0.0001**
Non-HGSC	156	5	151		11	145	
Stage							
I	107	11	96	0.5460	21	86	0.6050
II - IV	123	16	107		20	103	
Personal history of breast cancer^a^							
Diagnosed with breast cancer	6	2	4	0.1480	3	3	0.0714
Not diagnosed with breast cancer	224	25	199		38	186	
One or more family members with breast cancer^a,d^							
Present	26	5	21	0.3260	6	20	0.4040
Absent	154	17	137		25	129	
One or more family members with ovarian cancer^a,d^							
Present	13	6	7	**0.0017**	7	6	**0.0019**
Absent	167	16	151		24	143	
One or more family members with ovarian or breast cancer^a,c^							
Present	32	7	25	0.0775	9	23	0.1180
Absent	148	15	133		22	126	

^a^Among 230 patients, a detailed family history was available for 180 patients.

^b^Fisher's exact test.

^c^HGSC, high-grade serous carcinoma.

^d^Family history of cancer, including first- or second-degree relatives.

The frequency of histological OC subtypes among our patient cohort differed from that in Caucasian patients [4–7]; thus, we studied the association between the histological subtypes and pathogenic germline variants in our cohort. Although the major histological subtype of *BRCA1*/*2*-associated tumors was HGSC (22/27 cases, 81.5%), pathogenic germline *BRCA1*/*2* variants were also found in patients with other subtypes including endometrioid and clear cell carcinomas. The prevalence of pathogenic germline variants of *BRCA1*/*2* was much higher in patients with HGSC (22/74 , 29.7%) than in those with clear cell carcinoma (2/71, 2.8%) or endometrioid carcinoma (2/58, 3.4%).

In some Asian countries including Japan, genetic counseling and genetic testing have hitherto been offered to patients with OC and a family history of OC or breast cancer [11]. In our cohort, the frequencies of family history of HBOC-related and Lynch syndrome-related cancers were similar between pathogenic germline mutation-positive cases and negative cases (Supplementary Table 2). Therefore, we next focused on OC and breast cancer for family history analysis. Among 180 patients with available detailed family history, 32 had first or second-degree relatives with OC or breast cancer. Carriers of pathogenic germline *BRCA1/2* variants and any OC-associated gene variants were more likely to have first or second-degree relatives with OC (*P* = 0.0017 and 0.0019, respectively; Fisher's exact test). Individuals with OC and a personal history of breast cancer tended to carry pathogenic germline variants of *BRCA1/2* or other OC-associated genes, although the result did not reach statistical significance owing to the small number of such individuals available to be studied.

Descriptive statistical analysis showed that younger age, HGSC, and OC family history were significantly associated with positive pathogenic variant status of either *BRCA1/2* or any genes tested (Table 3). Therefore, logistic regression analysis was applied to model the relationship between these variables and pathogenic germline variants of *BRCA1/2* or any tested genes. After adjustment for these variables, only the HGSC subtype and OC family history remained as independent predictive factors for pathogenic germline *BRCA1/2* variants, whereas all 3 factors remained as independent predictive factors for pathogenic germline variants of any tested genes (Table 4). Because a detailed family history was not obtained from 50 of the 230 subjects, we also examined the association between variables excluding family history and pathogenic germline variants. By applying multiple logistic regression with stepwise variable selection, using *P* values as a selection criterion, the HGSC subtype and personal history of breast cancer remained as independent predictive factors for both pathogenic germline variants of *BRCA1/2* and any tested genes (Supplementary Table 3).

**Table 4:** Multivariate analysis to determine predictive clinicopathological factors of pathogenic germline variants of *BRCA1/2* or any tested genes in 180 patients with OC and a detailed family history^a^

Variable	Pathogenic *BRCA1/2* variant			Pathogenic variant in any tested gene		
	Odds ratio	95% CI^b^	Adjusted *P* value^c^	Odds ratio	95% CI^b^	Adjusted *P* value^c^
Age						
<55 vs. ≥55	2.62	0.862–7.970	0.0893	3.48	0.13–9.31	**0.0129**
One or more family members with ovarian cancer^d^						
Present vs. absent	6.58	1.52–28.60	**0.0119**	5.22	1.3–21.00	**0.0201**
Histologic subtype of OC^e^						
HGSC vs non-HGSC	12.3	3.97–38.40	**<0.0001**	10.4	4.05–26.80	**<0.0001**

^a^Multiple logistic regression was conducted using 3 variables that showed significant correlations with the mutations presented in Table 2.

^b^CI, confidence interval.

^c^Bold face text denotes statistically significant results.

^d^Family history of cancer including first- or second-degree relatives.

^e^HGSC, high-grade serous carcinoma.

## DISCUSSION

In this study, we performed TPS using a multigene panel to estimate the frequency of pathogenic germline variant carriers among Japanese patients with OC. Because this was not a large study, selection bias may have affected the study outcomes. However, the prevalence of pathogenic germline *BRCA1/2* variants in unselected Japanese patients with OC (27/230, 11.7%) did not show a large difference compared with that in other ethnicities, regardless of a large difference in prevalence of clear cell carcinoma, which showed a low frequency of pathogenic germline *BRCA1/2* variants, between Caucasian and East Asian populations (including Japanese) [8, 12–22]. Pathogenic variants in OC-associated genes other than *BRCA1/2* were also detected in 14 patients: 6 in 4 MMR genes and 8 in 5 other OC-associated genes. These pathogenic variants were also frequently observed in HGSC (8/74, 10.8%) compared with clear cell (2/71, 2.8%) and endometrioid (2/58, 3.4%) carcinomas. Because all genes with pathogenic variants in patients with OC except *GABRA6* are associated with DNA repair functions, individuals carrying pathogenic germline variants in DNA repair genes appear to be at risk for OC. Although the prevalence and penetrance of pathogenic germline variants differed among the DNA repair genes and the clinical utility of interventions in individuals with moderate-penetrance gene variants associated with OC risk remains unknown [23], multigene panel-based genetic testing, rather than single-gene testing, is an alternative tool for screening hereditary OC that enables more accurate genetic counseling [24].

Prediction of inherited risk in patients with OC is crucial for selecting patients who should be offered genetic risk evaluation by multigene panel-based genetic testing. As an independent risk variable, the age at OC diagnosis had not been generally associated with the likelihood of harboring an inherited pathogenic variant or with the gene in which a pathogenic variant was found [4]. In the patients with OC among our cohort, however, younger age (<55 years old) at the time of diagnosis was associated with positive pathogenic variants of *BRCA1/2* or any of the other tested genes. In terms of familial history, in addition, individuals with any first- or second-degree relatives with OC were associated with positive pathogenic variants of *BRCA1/2* or any of the other tested genes in the present study, although most individuals with positive pathogenic variants of *BRCA1/2* (15/22, 68.2%) or any of the other tested genes (22/29, 71.0%) did not have a family history of breast cancer/OC. Data in several previous reports also demonstrated that patients with OC and pathogenic germline *BRCA1/2* variants frequently lack a family history of breast cancer/OC [4, 13, 17]. Pathogenic germline *BRCA1/2* variants may predispose women to OC, particularly HGSC, through defective homologous recombination repair (HRR) function. Consistent with this hypothesis, pathogenic germline variants in other HRR-related genes including *PALB2*, *RAD51*, *RAD50*, *BARD1*, *CHEK2*, and *BRIP1* have been previously detected in serous OC [4, 25, 26]. Thirdly, in our study, patients with OC classified as HGSC were associated with positive pathogenic variants of *BRCA1/2* or any of the other tested genes.

Multiple logistic regression using these 3 variables revealed that the most significant predictors for germline variants in *BRCA1/2* or any of the other tested genes were the HGSC subtype and family history of OC. Predictive significance of the serous histologic subtype for pathogenic *BRCA1*/*2* variant-positive patients with OC was also reported in another Asian population [11]. Identifying pathogenic germline variant carriers among patients with OC will enable appropriate genetic counseling and will also enable these patients, especially those with pathogenic *BRCA1*/*2* variant-positive OC, to benefit from targeted therapy. However, pathogenic germline variants of *BRCA1*/*2*, other HRR-related genes, and MMR genes were also observed in non-HGSC subtypes (Table 3). Taken together, these data indicate that all patients with OC may be eligible for a cost-effective multigene testing using TPS, although those with inherited pathogenic variants could be predicted according to their clinicopathological features, such as age, family history of OC, and histologic subtype.

Individuals with pathogenic variants in MMR genes are predisposed to Lynch syndrome; moreover, OC is a Lynch syndrome-related cancer [27]. Among the MMR genes *MLH1*, *MSH2*, *MSH6*, and *PMS2*, defects in *MLH1* and *MSH2* account for most cases of Lynch syndrome and predominate in colon cancer [28]. In the present study, however, pathogenic variants were more frequently observed in *MSH6* and *PMS2* compared with *MLH1* and *MSH2* (Table 1). A similar finding was reported in a cohort of 1,915 unselected patients with OC that included 1,681 Caucasians: pathogenic variants were observed in *MSH6* in 3 cases and in *PMS2* in 4 cases, in contrast to 1 case with an *MLH1* pathogenic variant and no cases with a pathogenic variant in *MSH2* [28]. Collectively, *MSH6* and *PMS2* among the MMR genes appeared to be more strongly associated with OC compared with *MLH1* and *MSH2*, irrespective of ethnicity. Notably, OC in all cases with pathogenic germline *PMS2* variants in the present study (2/2 cases) and in a previous study (4/4 cases) [7] belonged to the HGSC subtype, whereas OC in some pathogenic germline *MSH6* variant cases in the present study (1/2 cases) and a previous study (2/3 cases) [7] was classified as endometrioid carcinoma. Therefore, *MSH6* and *PMS2* might be associated with different histologic subtypes of OC.

Several limitations of this study should be noted. The frequency of pathogenic variants may have been underestimated in this series of patients because we included only variants with a clearly damaging impact on protein function. Although we evaluated *BRCA1/2* variants according to a database from Myriad Genetics [9], some not-yet-characterized missense variants may prove damaging in OC-associated genes other than *BRCA1/2*. The frequency of patients with a personal history of breast cancer and a family history of various cancers may also have been underestimated, considering that we used samples from our biobank (KWB) and that it is difficult to obtain recent personal and family histories from patients who died or who were not followed-up in our hospital. A limited statistical power owing to the relatively small size of our cohort may also have prevented detection of the true association between pathogenic germline variants and clinicopathological features. In addition, the absence of an association does not necessarily imply the absence of a causal relationship between genetic predisposition and clinicopathological factors in patients with OC. Further studies are needed to clarify the prevalence and relevance of pathogenic germline variants in Japanese patients with OC.

In summary, our data suggest that the prevalence of pathogenic *BRCA1/2* variants and all tested OC-associated genes in Japanese patients with OC is similar to that in other ethnic groups, and that at least the HGSC subtype and OC family history may be useful for predicting the risk of genetic predisposition of Japanese patients with OC and referring high-risk patients for genetic counseling and testing.

## MATERIALS AND METHODS

### Study subjects and genomic DNA extraction

This study was approved by the Keio University School of Medicine Ethics Committee. All patients provided written, informed consent.

In total, 230 patients with OC treated in Keio University Hospital from 2001 to 2015 were enrolled for this analysis (Table 1). This study excluded the 102 patients with OC who had been recruited for our previous study, in which reported the correlation between family history and *BRCA1/2* status [29].

The histological subtypes included high-grade serous (*n* = 74), low-grade serous (*n* = 3), endometrioid (*n* = 58), clear cell (*n* = 71), mucinous (*n* = 18) and other cancers (*n* = 6). Histological diagnosis was performed by 2 independent pathologists and confirmed by 1 pathologist who specializes in gynecologic oncology (H.T.). Clinicopathological factors including familial cancer histories were collected and regularly updated through follow-up and questionnaires.

Germline DNA was isolated from whole blood samples using the QIAamp DNA Blood Kit (Qiagen, Hilden, Germany) and stored in the KWB. DNA concentrations were measured using a Qubit^®^ instrument (Thermo Fisher Scientific Inc., Waltham, MA, USA).

### Library construction, hybridization, and massively parallel sequencing

For TPS, 200 ng of genomic DNA from each patient was sheared using a Covaris S220 System sample-preparation instrument (Covaris, Woburn, MA, USA). After assessing the quality of the sheared DNA using a Bioanalyzer (Agilent Technologies, Santa Clara, CA, USA), paired-end libraries were prepared and hybridized with a custom pool of oligonucleotides targeting 75 or 79 genes (Supplementary Table 1) using the SureSelect XT Target Enrichment System (Agilent Technologies) according to the manufacturer's protocols. Following capture, samples were pooled for multiplexed sequencing and sequenced with 2 × 150 bp paired-end reads on a MiSeq (Illumina, San Diego, CA, USA).

### Bioinformatics analysis

Sequenced reads were mapped to the human genome reference (hg19) and SNVs, InDels, and CNVs were detected using SureCall Software v2.1/v3.5 (Agilent Technologies) and our pipeline for NGS data analysis, as described elsewhere [30–32], with a minor modification owing to a software update specific for a bioinformatics pipeline [32]. Minor-allele frequency data were referenced using the 1000 Genomes Project Database (http://www.1000genomes.org/), the NHLBI GO Exome Sequencing Project (ESP6500, http://evs.gs.washington.edu/EVS/), the Human Genetic Variation Database (HGVD, http://www.genome.med.kyoto-u.ac.jp/SnpDB/), and the Integrative Japanese Genome Variation Database (iJGVD, https://ijgvd.megabank.tohoku.ac.jp/). Pathogenic or likely pathogenic variants were validated by Sanger sequencing.

The detected variants in *BRCA1/2* were interpreted using a variant classification program according to the ACMG recommendations [10] and the database of Myriad Genetic Laboratories (Salt Lake City, UT, USA), which were developed with supporting linkage, biomedical, clinical, functional, and statistical data used for specific missense and intronic alterations based on over a million samples tested [9].

### Statistical analysis

Statistical analysis was performed using Prism 7 (GraphPad Software, La Jolla, CA, USA) or the R software package (version 3.3.2, https://www.r-project.org/). Fisher's exact test was performed to identify associations between categorical variables. For statistical purposes, family history of breast or ovarian cancer was limited to the first- and second-degree relatives only. A subset of variables, which showed a significant association (*P* < 0.05), or all variables, which were used for stepwise variable selection using *P* values as selection criteria, were studied by multivariate analysis to calculate the logarithm of odds of carrying pathogenic germline variants, using logistic regression. All statistical analyses were two-sided, and *P* values < 0.05 were considered statistically significant.

## SUPPLEMENTARY MATERIALS TABLES


