# Supplementary material for: Prevalence of pathogenic germline variants detected by multigene sequencing in unselected Japanese patients with ovarian cancer
Source: Oncotarget. 2017 Nov 28;8(68):112258–67. doi: 10.18632/oncotarget.22733 (PMC5762508; doi:10.18632/oncotarget.22733)
Supplement: Supplementary file 1 [file oncotarget-08-112258-s001.pdf]

Prevalence of pathogenic germline variants detected by multigene sequencing in unselected Japanese patients with ovarian cancer

SUPPLEMENTARY MATERIALS

Supplementary Table 1: List of 79 genes analyzed by targeted resequencing

AKT1	*BRIP1*	*CSMD3*	*FANCL*[*]	*IGF2R*	*MRE11A*	*PALB2*	*PTEN*	*SMAD4*
APC	*CBLC*	*CTNNB1*	*FAT3*	*KIT*	*MSH2*	*PDGFRA*	*RAD50*	*SMARCA4*
ARID1A	*CCNE1*	*CUBN*	*FGFR1*	*KRAS*	*MSH3*	*PIK3CA*	*RAD51B*[*]	*STK11*
ATM	*CDH1*	*EGFR*	*FGFR2*	*KREMEN1*	*MSH6*	*PIKR1*	*RAD51C*	*TGFBR2*
BARD1	*CDK12*	*EMSY*[*]	*GABRA6*	*MAS1L*	*MUTYH*	*PMS2*	*RAD51D*	*TP53*
BMPR1A	*CDK4*	*EPCAM*	*GNAS*	*MLH1*	*MYC*	*POLD1*	*RAD54L*[*]	*USP16*
BRAF	*CDKN2A*	*ERBB2*	*GREM1*	*MLH3*	*NBN*	*POLE*	*RB1*	*XRCC2*
BRCA1	*CHEK1*	*FAM175A*	*HNF1A*	*MLL2*	*NF1*	*PPM1D*	*RECQL*	
BRCA2	*CHEK2*	*FANCC*	*HNF1B*	*MLL3*	*NRAS*	*PPP2R1A*	*SMAD2*	

[*]Genes (4) excluded from analysis in 44 samples.

Supplementary Table 2: Correlation between pathogenic germline variants and family histories

Family history with first- or second-degree relatives	Pathogenic germline variants in any tested gene				
	BRCA1/2	**MMR genes**[a]	**Other genes**[b]	**Any gene positive**	**All negative**
HBOC-related cancers[c]	8/22 (36.4%)	1/3 (33.3%)	3/6 (50%)	12/31 (38.7%)	44/149 (29.5%)
Lynch syndrome-related cancers[d]	10/22 (45.5%)	0/3 (0%)	4/6 (66.7%)	14/31 (45.2%)	63/149 (49.0%)

[a]*MLH1, MSH2, MSH6,* and *PMS2*.
[b]*RAD51D, ATM, MRE11A, FANCC,* and *GABRA*.
[c]HBOC (hereditary breast and ovarian cancer)-related cancers included breast, ovarian, pancreatic, and prostate cancers.
[d]Lynch syndrome-related cancers included colorectal, endometrial, gastric, ovarian, pancreatic, ureter, renal pelvis, biliary tract, brain duct, brain, and small intestinal cancers, as well as sebaceous gland adenomas and keratoacanthomas.

Supplementary Table 3: Multivariate analysis to determine predictive clinicopathological factors of pathogenic germline variants of *BRCA1/2* or any tested genes in 230 patients with OC[a]

Variable	Pathogenic *BRCA1/2* variant			Pathogenic variant in any tested gene		
	**Odds ratio**	**95% CI**[b]	**Adjusted *P* value**[c]	**Odds ratio**	**95% CI**[b]	**Adjusted *P* value**[c]
Personal history of breast cancer						
Present vs. absent	11.80	1.43–96.90	**0.0219**	11.7	1.91–72.00	**0.0079**
Histologic subtype of OC[d]						
HGSC vs non-HGSC	15.70	5.17–47.70	**<0.0001**	10.60	4.69–24.00	**<0.0001**

[a]Multiple logistic regression with stepwise variable selection using *P*-values as selection criteria was performed with all variables in Table 3, except for family history.
[b]CI, confidence interval.
[c]Bold face text denotes statistically significant results.
[d]HGSC, high-grade serous carcinoma.
